
JOURNAL INFORMATION
==============================
NLM Title Abbreviation: ScientificWorldJournal
Journal ID: ScientificWorldJournal
NLM Journal ID: 101131163
Journal ID: TSWJ

The Scientific World Journal

ISSN: 2356-6140
EISSN: 1537-744X
Publisher: Wiley

ARTICLE INFORMATION
==============================
PMCID: PMC6084719
DOI: 10.1100/tsw.2001.407
Article ID: 903475
Article version: 1
Subjects: Extended Abstract

CGRP and Adrenomedullin Modulate Synaptic Transmission in Purkinje Cells

Cater H.
Poyner D.
Hartell N.
Pharmaceutical Sciences Research Institute, Aston University, Birmingham, England
Publication date: 2001
Electronic publication date: 2001 Nov 9
Volume: 1
First page: 37
Last page: 37
Copyright: Copyright © 2001 H. Cater et al.
Copyright year: 2001
License: This is an open access article distributed under the Creative Commons Attribution License, which permits unrestricted use, distribution, and reproduction in any medium, provided the original work is properly cited.
License URL: https://creativecommons.org/licenses/by/3.0/

==============================
4th International Meeting on Calcitonin Gene-Related Peptide (CGRP)
*TheScientificWorld* (2001) 1(S1), 37
ISSN 1532-2246; DOI 10.1100/tsw.2001.407

### CGRP AND ADRENOMEDULLIN MODULATE SYNAPTIC TRANSMISSION IN PURKINJE CELLS

H. Cater, D. Poyner, and N. Hartell
Pharmaceutical Sciences Research Institute, Aston University, Birmingham, England

Binding studies have demonstrated the presence of calcitonin gene-related peptide (CGRP) receptors in the cerebellum. There has been little work on the electrophysiology and pharmacology of CGRP in the cerebellum. We have assessed the effect of CGRP, adrenomedullin, and the antagonist $CGRP_{8-37}$ on synaptic transmission between parallel fibres (PFs) and Purkinje cells (PCs) and on spontaneous synaptic events (mEPSCs).

Sagittal cerebellar slices (200-μm thick) were obtained from rats and whole-cell patch clamp recordings were made from PCs whilst stimulating PFs. The 10-min application of 50 and 10 nM CGRP significantly decreased EPSC amplitude (50 nM 76.95 ± 7.11%, n = 10; 10 nM 77.38 ± 7.11%, n = 8; $p < 0.01$). The effect of 10 nM CGRP was inhibited with 5-min preapplication of 1 μM $CGRP_{8-37}$ (106.03 ± 9.29%, n = 6, $p < 0.01$) but not with 100 nM $CGRP_{8-37}$ (79.91 ± 5.61%, n = 7). However, 10 nM $CGRP_{8-37}$ alone significantly decreased EPSC amplitude similar to CGRP (48.34 ± 6.55%, n = 5, $p < 0.01$). In contrast, 50 nM adrenomedullin significantly increased EPSC amplitude (143.58 ± 24.5%, n = 7, $p < 0.01$).

10 nM CGRP increased the paired pulse ratio (105.43 ± 4.95%, n = 6) indicating the effect could be presynaptic. 50 nM CGRP significantly decreased the frequency of spontaneously evoked mEPSCs (62.4 ± 7.4%, n = 5, $p < 0.01$) but did not affect the amplitude.

This demonstrates that CGRP modulates EPSC responses following PF stimulation of PCs and can be blocked by $CGRP_{8-37}$. This effect is probably through CGRP receptors rather than through adrenomedullin receptors and is likely to be presynaptic
